# Transient bio-inspired gliders with embodied humidity responsive actuators for environmental sensing

**DOI:** 10.3389/frobt.2022.1011793

**Published:** 2022-10-31

**Authors:** Fabian Wiesemüller, Ziwen Meng, Yijie Hu, Andre Farinha, Yunus Govdeli, Pham H. Nguyen, Gustav Nyström, Mirko Kovač

**Affiliations:** ^1^ Materials and Technology Center of Robotics, Department of Functional Materials, Empa–Swiss Federal Laboratories for Materials Science and Technology, Dübendorf, Switzerland; ^2^ Aerial Robotics Laboratory, Department of Aeronautics, Imperial College London, London, United Kingdom; ^3^ Laboratory for Cellulose & Wood Materials, Department of Functional Materials, Empa–Swiss Federal Laboratories for Materials Science and Technology, Dübendorf, Switzerland; ^4^ State Key Laboratory of Pulp and Paper Engineering, South China University of Technology, Guangzhou, China; ^5^ Department of Health Sciences and Technology, ETH Zürich, Zürich, Switzerland

**Keywords:** biodegradable materials, transient robotics, environmental sensing, aerial robotics, bio-inspiration

## Abstract

Collecting temporal and spatial high-resolution environmental data can guide studies in environmental sciences to gain insights in ecological processes. The utilization of automated robotic systems to collect these types of data can maximize accuracy, resilience, and deployment rate. Furthermore, it reduces the risk to researchers deploying sensors in inaccessible environments and can significantly increase the cost-effectiveness of such studies. The introduction of transient robotic systems featuring embodied environmental sensors pushes towards building a digital ecology, while introducing only minimal disturbance to the environment. Transient robots made from fully biodegradable and non-fossil based materials, do not develop into hazardous e-waste at the end of their lifetime and can thus enable a broader adoption for environmental sensing in the real world. In this work, our approach towards the design of transient robots includes the integration of humidity-responsive materials in a glider, which is inspired by the *Alsomitra macrocarpa* seed. The design space of these gliders is explored and their behavior studied numerically, which allows us to make predictions on their flight characteristics. Results are validated against experiments, which show two different gliding behaviors, that can help improve the spread of the sensors. By tailoring the Cellulose-Gelatin composition of the humidity actuator, self-folding systems for selective rainwater exposure can be designed. The pH sensing layer, protected by the actuator, provides visual feedback on the pH of the rainwater. The presented methods can guide further concepts developing transient aerial robotic systems for sustainable, environmental monitoring.

## 1 Introduction

With the advent of robotic systems, researchers have gained access to a variety of tools enabling the automated collection of samples, deployment of sensors and measurements of environmental parameters. Therefore, personnel costs, as well as potential dangers to the workers deploying those sensors or collecting samples, can be reduced. However, if such robotic systems break or malfunction they can end up as potentially toxic e-waste and be a threat to the environment, especially in sensitive habitats. By manufacturing the environmental sensing robot from biodegradable and non-toxic materials, the systems can be deployed for one-way missions as introduced by Wiesemüller et al. (2021) and Sethi et al. (2022). In detail, such transient robotic systems can be deployed in nature, to collect data and at the end of their service life biodegrade and leave no waste behind. Additionally, the nutritional sources stored in a robot’s body made from non-fossil biopolymers is brought back into the nutrition cycle and enable a circular approach towards robotic data retrieval as discussed by Mazzolai and Laschi (2020), Hartmann et al. (2021) and Winfield and Ieropoulos (2016).

To enable low-powered, yet biodegradable deployable aerial systems, the design of seeds that utilize flight as their means of seed dispersal was investigated. Aerial seeds, utilize gliding and spiraling, to travel large distances through the exchange of potential energy (from altitude change) for kinetic energy Nathan et al. (2002), Jones (1995). The investigation of the physical intelligence Ortega Ancel et al. (2017) embedded within the gliding mechanics of aerial seeds, enables the design of highly multi-modal and morphologically adaptive flyers with unique flight capabilities and improved flight efficiencies. Plant seeds also have morphological adaptations to assist with glide or parachuting to assist with wind seed dispersal. The asian climbing gourd (e.g., *Alsomitra macrocarpa*), western salsify (*Tragopogon dubius*), box elder (*Acer negundo*), bigleaf maple (*Alsomitra macrocarpa*), evergreen ash (*Fraxinus griffithii*), tipa Tipuana (*Tipuana tipu*), spinning fruit (*Gyrocarpaceae*) seeds, etc. are some among the long list of plant seeds that are able to glide, spin and glide, or parachute through the air Nathan et al. (2002); Jones (1995). There have been various robotics literature inspired by plant seeds. Among which includes studies of wind dispersal seed to create micro-gliders Kim et al. (2021), the study of self-burial found in seeds of filaree Erodium cicutarium (Geraniaceae) Evangelista et al. (2011), and other samara inspired gliders Pounds and Singh (2015). Unlike this work, previous samara inspired gliders do not look into the transient nature and flexible properties of the samara seed wings.

**FIGURE 1:**
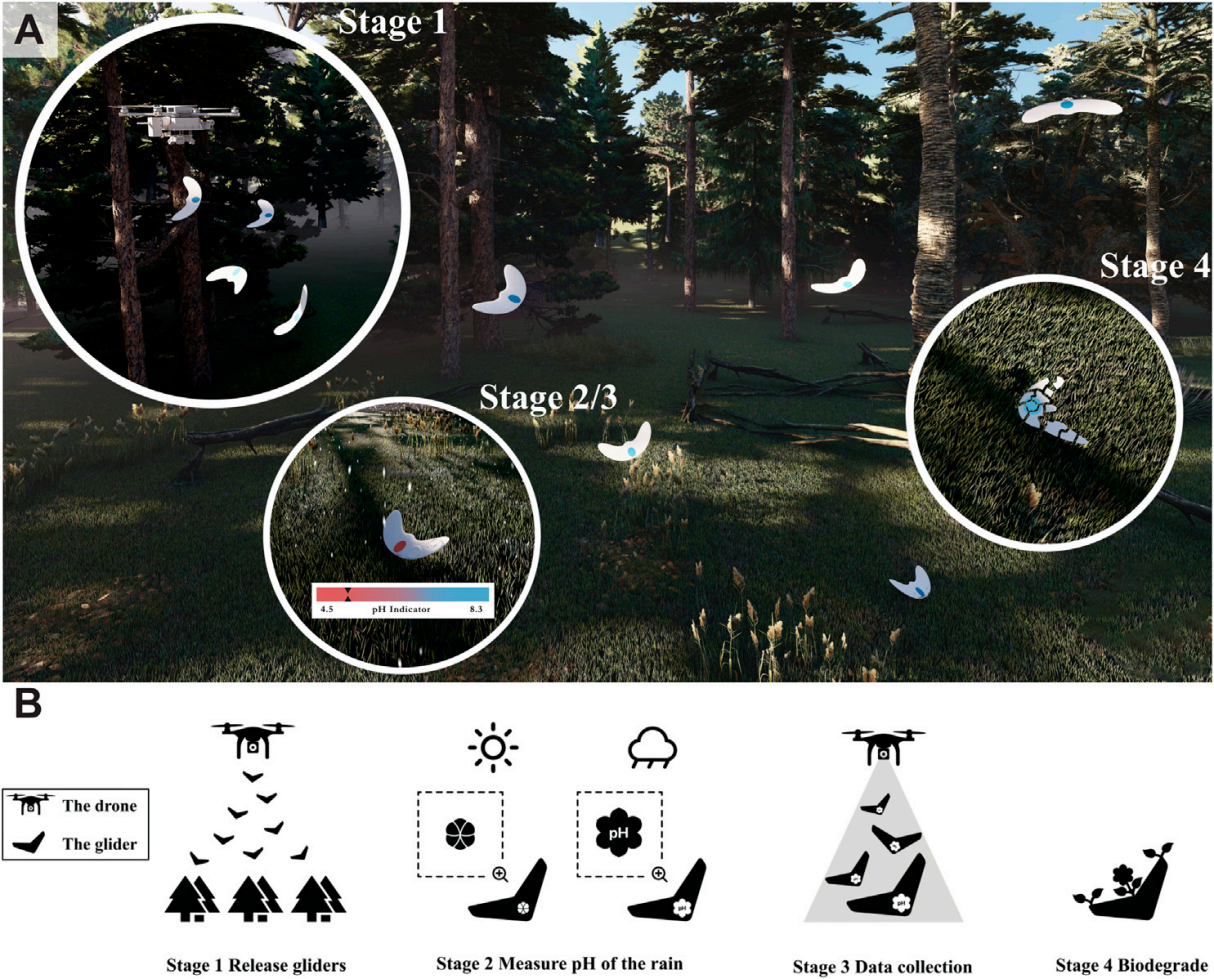
Overview of the proposed transient glider deployment in nature. **(A)** Conceptual illustration of the robot’s deployment (stage 1), the pH detection and data collection (stage 2/3) and the glider’s biodegradation (stage 4), **(B)** Mission profile of the transient robot. After release by a UAV, the gliders land on the ground. When exposed to rain, the humidity actuator opens up and exposes the embodied pH sensor to the potentially acid rain. The color change corresponds to the acidity of the rain water.

Acid rain with pH from 4.2 to 4.4 is one of the well-known threats to plants and soil quality in forests Burns et al. (2016). Herein, by integrating litmus–a pH reactive biodegradable chemical, which changes color from blue to red when exposed to acids - bio-inspired gliders fitted with humidity actuators, autonomous environmental sensing and spatial monitoring transient robots are designed for detecting the pH of rainwater. The conceptual illustration in Figure 1 highlights the envisioned full mission profile, in which a multicopter carrying several of the gliders follows a predefined trajectory and deploys them automatically. After the glider has landed on the ground or canopy of the trees, the pH sensor is covered with a protective layer. When the protective layer, manufactured from a moisture responsive material, gets in contact with rain water, opens up and exposes the sensing area to the ambient air. Hence, water droplets targeted for analysis can get in contact with the Litmus patch and induce a color change based on the pH. After the rainfall has stopped, the deployment drone comes for a fly-by to visually detect the color change and thus give an approximate pH value. By repeating the measurement with the other deployed gliders a spatial map can be build up corresponding to the local varieties of the pH of the rainwater. After the data is retrieved, the gliders are left for biodegradation, without leaving any e-waste behind.

To protect the pH sensor and reduce the effect on it by the ambient atmospheric conditions during storage, a humidity-responsive actuator is placed over the sensing area. This actuator is required to open when exposed to rain-water. In literature, various approaches to manufacture such actuators are discussed by Ma et al. (2013), Arazoe et al. (2016), Li et al. (2021) and Ji et al. (2014). For example, Mu et al. (2018) took advantage of the nanoscale molecular channels and vapor-absorbing functional groups present in Nafion, which is a commercial perfluorosulfonic acid ionomer film, to fabricate vapor-driven actuators. Since exercise increases human perspiration levels, the actuators appeared to enable venting between the skin and the external environment, thereby facilitating control of body moisture and skin temperature. Wei et al. (2021) fabricated a cellulose nanofibers/carbon nanotube/graphene oxide composite film-based humidity actuator *via* an efficient vacuum-assisted self-assembly method. The film could serve as mechanical grippers to grasp a plastic foam by applying water vapor *via* the plastic straw, and to release the foam when the water vapor is removed. However, all these actuators only bend in the opposite direction from where the vapor is coming from. In our case, the actuator has to bend towards the side with the higher humidity to expose the pH sensor underneath. Additionally, the actuator needs to be manufactured from biodegradable and non-fossil materials. In order to achieve the goal, a bi-layer humidity actuator was designed by using cellulose-nanofiber-gelatin composite film (CNF:G) as an active layer and shellac as a passive layer. Shellac is a nontoxic natural animal secretory substance. It can be dissolved in alcohol to make liquid shellac, and seals out moisture as discussed by Zhang et al. (2020) and Luangtana-anan et al. (2017). With rain drops falling on the actuator, the actuator will open and therefore the pH sensor can detect the pH information of the rainwater. Various CNF:G compositions were investigated to tune the humidity sensitivity, degradability and mechanical properties as indicated by Campodoni et al. (2020).

## 2 Materials and methods

### 2.1 Bio-inspired glider

To approach the task of designing bio-inspired, lightweight and small sized gliders, that could carry an approximate payload of 0.5–2 g and vary its gliding range, when launched from a multicopter, the Javan Cucumber seed (*Alsomitra Macrocarpa*) was uses as an initial template. By nature, the Javan Cucumber seed highlights the lowest rate of descent with a high wing loading Minami and Azuma (2003), capable of gliding at a descent rate of 0.3–0.7 m s^−1^ Azuma and Okuno (1987). The Javan cucumber seed also has inspired designs of fixed-wing air crafts in the past Bishop (1961). Some of the aerodynamic design features found in the Javan Cucumber seed are that the center of gravity of the seed is just in front of the seed location on its body, its thickness varies from 1 mm to a sub-millimeter thickness, it also utilizes a swept and tapered plan form, features a reflexed trailing edge, and has a twisted washout Azuma and Okuno (1987), Minami and Azuma (2003).

In previous work, for such thin aerial gliders, like the butterfly wing Ortega Ancel et al. (2017) or rotary seed Kim et al. (2021), often simplify their airfoils to be a flat plate for both the manufacturing and the aerodynamic studies. The design variations are often predicted based on different shape designs rather than a complete geometrical design optimization scheme. Although an airfoil profile is provided by Azuma and Okuno (1987), difficulties in designing a self-stabilizing airfoil based on it (from early tests with XFLR5) were encountered. Thus, our design starting point utilized a self-stabilizing Fauvel airfoil Prisacariu (2018), which was then geometrically modified to match the Javan cucumber seed’s shape, as seen in Figure 3A. In this manner, the camber of the flying seed is also accounted for.

To model the geometry of the Javan cucumber with adaptive design parameters such as wing sweep, taper, dihedral, wash-in/out, and center of gravity (CG) location, XFLR5 was utilized as a starting point. The Javan cucumber seed shape and dimensions were referenced from Azuma and Okuno (1987), and the designs are shown in Figure 3A. The type 1 glider is designed based on the exact geometry obtained from the referenced literature, while the type 2 glider is modified based on the type 1 glider, with larger wing area at the expense of lower aspect ratio.

#### 2.1.1 Bio-inspired glider fabrication

**FIGURE 2:**
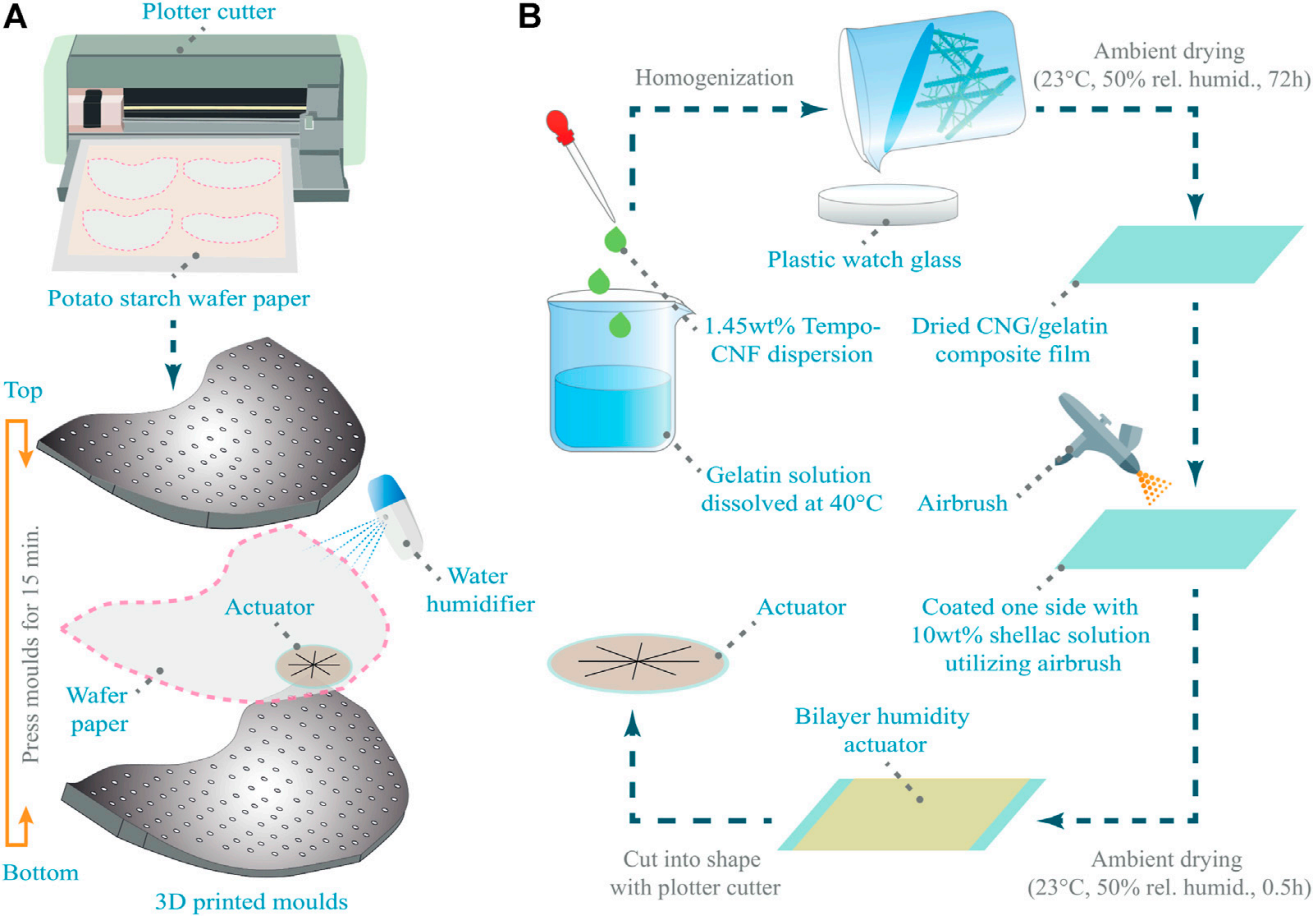
Manufacturing flowcharts of the glider and actuators. **(A)** The potato paper is cut using a plotter cutter. Afterwards the flat template is wetted, pressed into a 3D shape using a 3D printed mold and the actuator is bonded to the air-frame, **(B)** After the TEMPO-CNF dispersion is homogenized with the Gelatin solution, the mixture is poured on a glass plate and dried in ambient conditions. The dried films are then manually coated with 10wt% shellac solution. After drying the actuators are cut into the desired shape.

In order to fabricate the bio-gliders, the potato starch wafer paper is first aligned and cut utilizing a plotter cutter (Cameo 4, Silhouette America Inc., Lindon, Utah, United States), as seen in Figure 2A. Two-halves of the press molds are designed and 3D printed with 1.5 mm diameter holes to promote quicker drying. The wafer paper cutouts are sprayed evenly with a handheld water humidifier and sandwiched between the two-halves of the molds, pressed and clamped together with C-clamps. After an alloted wait time of 15 min, the bio-glider is taken out of the mold with the appropriate wing tip features seen in its shape. The manufactured humidity actuator, as shown in Figures 2B, is then bonded onto the glider at the correct position for the designed center of gravity, to complete the manufacturing of the bio-glider.

#### 2.1.2 CFD simulation setup

Computational Fluid Dynamics (CFD) simulations were conducted to investigate the influence of complex flow features present around the gliders, and the longitudinal forces which describe the overall performance of the gliders. For this purpose, the in-compressible Reynolds averaged Navier-Stokes (N-S) equations with decoupled pressure and momentum equations were solved. Furthermore, the micro gliders designed are shown, in the XFLR predictions, to operate at Re = [1.5, 5] ⋅ 10^4^, where turbulence is expected. Menter’s two equation turbulence model *k* − *ω* SST and the *γ* − *Re*_*θ*_ transition model closure are chosen to model both the turbulent flow but also the laminar-turbulent transition of the boundary layer. The N-S equations are solved until the wall and thus *y* ^+^ ∼ 1 is enforced at the first cell. The geometry preparation, analysis and post-processing are carried out in *StarCCM* ^+^2021.

The numerical domain was chosen such that relevant flow features such as the wake are fully captured. Advantage of the glider’s symmetry to model half it is wingspan (*l*) was taken. Furthermore, a rectangular domain of 0.75 *l* upstream and 7 *l* downstream length, 0.75 *l* width and 1 *l* height were chosen. An unstructured tetrahedral mesh is used to discretise the geometry and additional leading edge, trailing edge and wake refinements ensure sufficient resolution around interesting flow features. An inflation layer with 16 elements and growth rate of 1.3 is used to capture the boundary layer. A mesh convergence study is performed using 4 meshes for glider type 1 at 0° and 8° angle of attack (AoA). A discretisation error estimate is obtained by executing a least-squares fit for asymptotic convergence of lift, drag and moment coefficients as function of refinement ratios $r_{r}=\sqrt[{3}]{\frac{N_{1}}{Ni}}$ . The second most refined mesh was chosen based on the results shown in Supplementary Figure S1; Supplementary Table S1, which corresponds to approximately 6 million cells and takes approximately 290 s/CPU to run.

#### 2.1.3 Gliding experimental set-up

For the indoor tests passive reflective marker tapes are cut and attached onto both sides of the glider to track its motion and provide localization data. The flight tests are conducted in a 10 × 6.2 × 5.5 m flight arena, equipped with 16 Vicon tracking cameras (T40, Vicon Motion Systems Ltd., Oxford, United States), with millimeter accuracy. A gripper with a pan-tilt mechanism was used to control the angle at which the glider was being released, in order to represent the various conditions that could potentially happen when a drone is used for deployment. For the outdoor tests nine gliders have been launched five times each from a 30 cm long launching slope mounted on a 6.9 m high bridge at an angle of 45°. The landing site of the gliders was measured using a tape measure. During the testing crosswinds at the launching position were measured using a hot-wire anemometer (testo 405i, Testo SE & Co. KGaA, Titisee-Neustadt, Germany). The average cross-wind speed during testing was 0.53 m/sec and the 20 s gust-speed was found to be 2.4 m/sec.

### 2.2 Humidity reactive bio-polymer films

#### 2.2.1 Cellulose nanofibre (CNF) dispersion

0.1 mM (2,2,6,6-tetramethylpiperidin-1-yl)oxyl (TEMPO) and 1.0 mM NaBr per gram of cellulose pulp were mixed with 2 wt% cellulose fibre (elemental chlorine-free fibres extracted from bleached softwood pulp fibres, Mercer Stendal Company, Berlin, Germany) aqueous suspension. The pH is adjusted to 10 by using NaOH solution, 10 mM NaClO per gram of cellulose pulp was added for the cellulose oxidation. Afterwards, the TEMPO-oxidized cellulose fibers were washed until the conductivity was similar to that of distilled water. Finally, the oxidized and purified cellulose fibres aqueous suspensions were ground by a Supermass Collider (MKZA10-20J CE, Masuko Sangyo Co. Ltd., Kawaguchi, Japan) to obtain a stable CNF dispersion with a concentration 1.4 wt%.

#### 2.2.2 Humidity-responsive actuators

Typically, the CNF:G films are made by mixing gelatin solution with CNF suspension with a total solid content of 1 wt%. After stirring at low speed (to avoid inducing air bubbles) for 1 h, the mixture is poured on a plastic watch glass to a thickness of 6 mm and dried at room temperature. The film compositions are summarized in Table 1. Due to the induced non-planar deformation caused by residual stresses during drying, the 1 wt% CNF films were manufactured through vacuum filtration. Therefore, 0.65 *μm* membrane filters (Durapore DVPP 0.65 *μm*, Merck KGaA, Darmstadt, Germany) were used to filtrate the 1 wt% CNF suspension to form 30 *μm* thick films. The wet films were dried using a hot press (Sheet Former, Estanit GmbH, Mülheim an der Ruhr, Germany) at 90°C and 20 bar for 15 min to obtain the final CNF film. As illustrated in Figure 2B the actuator was manufactured by coating one side of the film with 10 wt% shellac solution by using an air brush. The bi-layer film was then dried and cut into desired shape by using a blade plotter (Cricut Maker, Cricut Inc., South Jordan, Utah, United States), and was stuck onto the glider using starch (Sobocat HC, Südstärke GmbH, Schrobenhausen, Germany) solution as a bonding adhesive. For characterization rectangular specimens (10 mm × 30 mm) were cut out. For the integration into the glider 35 mm diameter disks were cut out, and four 25 mm long cuts were added to form a star shaped pattern. Pictures of the closed actuator before and after spraying it with water are given in Figures 10E,F, respectively.

**TABLE 1:** Overview of the various compositions of the actuator films manufactured. Of each composition five samples were tested. Note that the films marked with an asterisk were manufactured through vacuum filtration.

Sample	Solid-content CNF [wt%]	Solid-content gelatin [wt%]	Thickness ± std [*μ*m]
G	0	1	32.4 ± 1.95
CNF:G	0.25	0.75	37.6 ± 1.14
CNF:G	0.50	0.50	36.2 ± 2.48
CNF:G	0.75	0.25	37.0 ± 0.10
CNF*	1	0	26.6 ± 0.89

## 3 Results

### 3.1 Bio-inspired glider

Using the shape of the Javan cucumber seed as a starting point, the flight performance and behavior of two different glider geometries were investigated using different simulation approaches. Indoor flight tests of the two glider geometries with different mass distributions are shown alongside the simulation results.

#### 3.1.1 Airfoil selection

**FIGURE 3:**
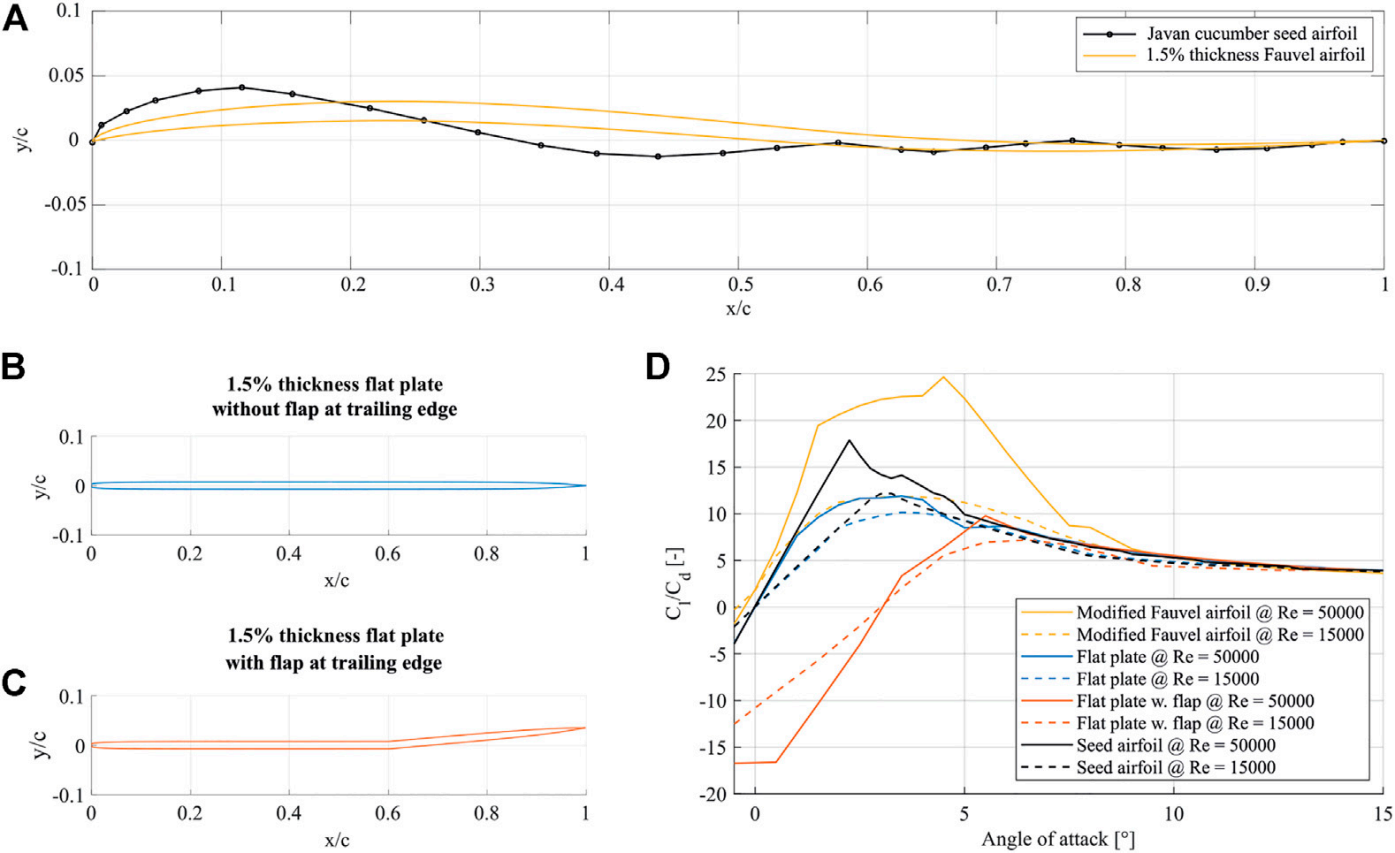
Side-view of the selected airfoils for the bio-inspired glider. **(A)** gives the airfoil height normalized by the chord-length versus the chord normalized by the chord-length of the modified Fauvel airfoil compared to the Javan cucumber airfoil according to Azuma and Okuno (1987), **(B)** gives the side-view geometry (normalized height versus normalized chord) of the investigated flat plate at 1.5% thickness, **(C)** gives the side-view geometry (normalized height versus normalized chord) of the investigated flat plate airfoil with a flap in the trailing edge and 1.5% thickness, **(D)** gives the ratio of lift to drag coefficients vs. AoA for the original seed airfoil, the flat plate airfoil, the flat-plate airfoil with a flap and the modified Fauvel airfoil at Re = 15*k* and Re = 50 *k*.

Javan cucumber seeds show large variation in shape and size. Depending on the seed type, the center of gravity, core position, wing-span and aspect ratio vary significantly. The average seed dimensions and weight in Azuma and Okuno (1987) are used as reference for the initial modeling work. The reflexed airfoil shape is shown in Figure 3A alongside the averaged cross-section of the seed. The final 44% of the seed chord length is irregular in thickness and shape, which is omitted from the design for two main reasons: Firstly, a reflexed airfoil should ideally produce a balancing moment close to the trailing edge for pitching up motion. However, the trailing edge of the seed does not produce this effect. Secondly, the contribution of this irregular shape to the gliding efficiency would be minimal if not adverse. The chosen airfoil is thus based on an auto-stable airfoil designed for flying wings by Charles Fauvel, reshaped for 1.5% thickness. The thickness was chosen to be as similar as possible to the wing profile of the Javan cucumber. It is shown in Figure 3D that the chosen reflex airfoil achieves higher lift-to-drag ratios than the seed airfoil for the selected AoA range, noting that the efficiency of the modified airfoil increases further with increasing Reynolds number.

A matter of concern that arises with the choice of a significantly complex airfoil shape is manufacturing complexity, as it is less demanding to manufacture and ensure shape consistency if gliders are designed from flat sections. The possibility of using simpler shapes of equivalent maximum thickness, such as a flat-like symmetric airfoil (Figure 3B) and a flat-like symmetric airfoil with a flap at 80% of the chord (Figure 3C), were investigated. Both of them are considered less favorable for the proposed application. As shown in Figure 3D, the symmetric airfoils cannot produce comparable lift force to the other two at higher Re, and the design with trailing edge flap has overall lower efficiency. In addition to the benefit of using Fauvel airfoil to lift-to-drag ratio, this type of airfoil can also help the glider to self-stabilize under trimmed flight conditions with the help of a reflexed camber line.

#### 3.1.2 Gliding performance

**FIGURE 4:**
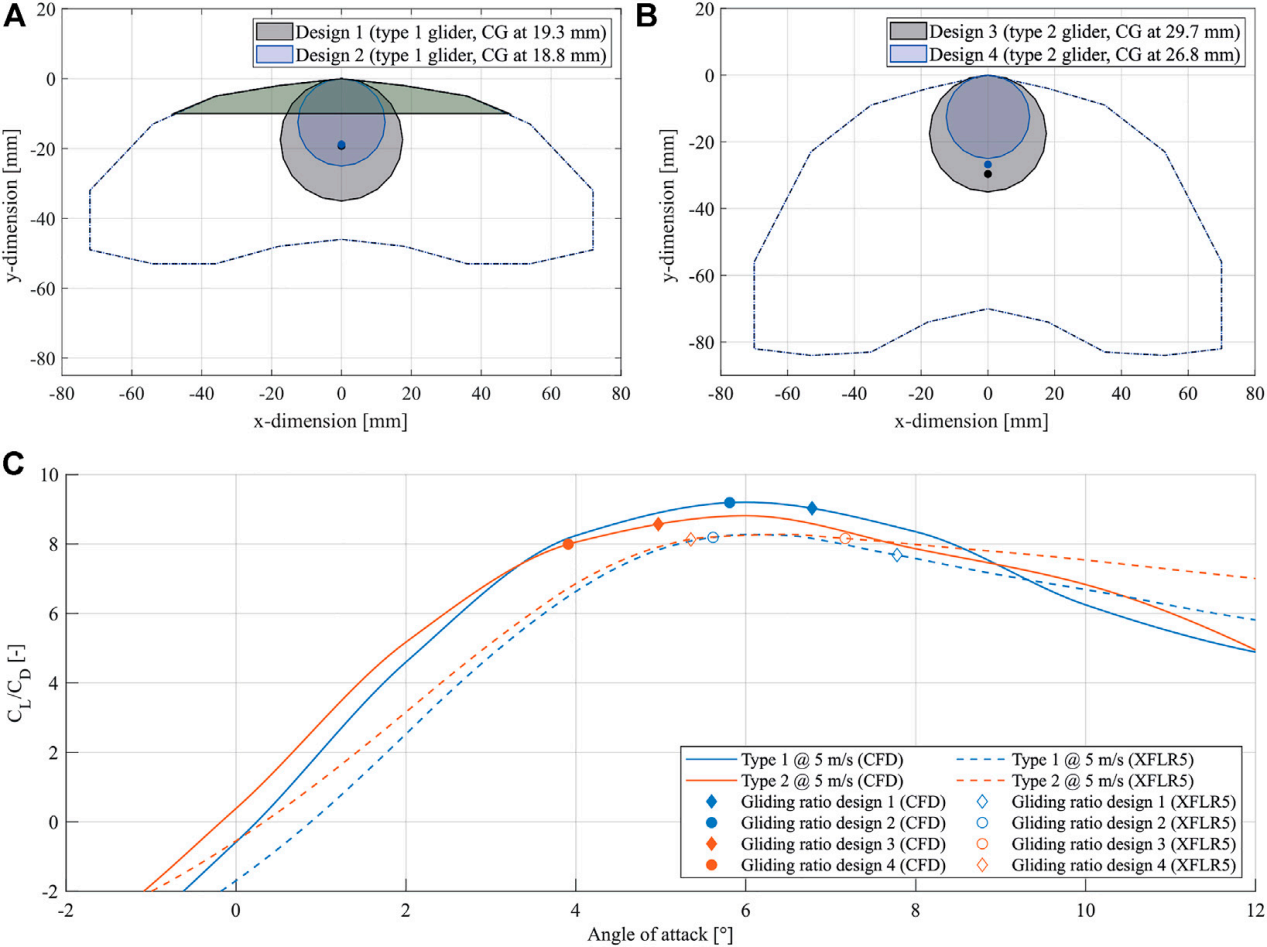
Overview of the glider’s shape and flight performance. **(A)** represents the slender wing of the glider type 1 with the two tested center of gravity indicated. The distance of the center of gravity from the leading edge is given. The light green area marks the location of additional weight that had to be added to achieve stable flight, **(B)** represents the wide wing of the glider type 2 with the two tested center of gravity marked. The distance of the center of gravity from the leading edge is given, **(C)** shows the *lift*/*drag* ratios versus angle of attack for different glider designs. The results using CFD and XFLR5 are given.

XFLR5 is used for initial design and exploration of parameters such as wing area ratio, dihedral and washout, due to its ease of use when designing and evaluating the aerodynamic qualities of aircraft. Candidate glider shapes obtained this way are then studied in terms of longitudinal flight characteristics and these results are compared to results obtained in CFD. This increases confidence in the results as the vortex lattice and 3D panel methods used by XFLR5 are generally more reliable at higher Reynolds numbers. Moreover, CFD simulations provide an in-depth visualization of separation bubbles on a surface, allowing for further refinement of the 3D glider shape. The final geometries used in analysis weigh between 1.25 and 1.75 g and are shown in Figures 4A,B where 25 mm and 35 mm diameter humidity actuators are placed near the leading edge to move the CG forward and improve longitudinal stability. An additional payload of 0.3 g is also added to the leading edge of design 1 and 2 to further improve stability.

Both lift and drag forces are shown to be higher in the CFD analysis than in XFLR5, particularly at lower AoA. However, both methods predict similar glide ratios, albeit at different AoA. The differences in estimated forces are not attributed to a particular flow feature found in CFD, and are likely a result of better accuracy in CFD simulations. The difference in gliding AoA is directly related to the strong disparity in the moment coefficient curves obtained with both methods and the resulting widely different stable AoA for a given CG position. The reason for this is the a small separation bubble that tends to form in the reflex portion of the chosen airfoil, which results in a more pronounced pitch-down moment at higher AoA and better stability than predicted in XFLR5. A figure of the flow fields at 8° and 10° AoA visualizing the separation bubble is given in the Supplementary Figure S2. A theoretical glide ratio can be extracted from the results shown in Figure 4C where XFLR results predict advantageous glide angles at AoA above 5°, while CFD expects an optimal range at 4°–8° to result in glide angles of 6°–7°.

Even if statically stable configurations have been found with XFLR5 and CFD, this alone is no guarantee of good gliding characteristics. By studying the dynamic stability of the gliders, one can better predict their trajectory, better evaluate their performance and directly compare the simulations with experimental free flight results. Longitudinal flight dynamics simulations were performed by solving the system of 6 equations formed by Eqs 1, 2, where the force terms *F*_*u*_, *F*_*w*_ and *M*_*v*_ are evaluated using the coefficients calculated from the CFD simulations, and *R* the rotation matrix that relates the glider’s state in the body frame of reference to the fixed frame of reference.

$$\left[\begin{array}{c} F_{u}\\ F_{w}\\ M_{v} \end{array}\right]={\left[\begin{array}{c} m\\ m\\ I_{yy} \end{array}\right]}_{\mathrm{diag}}\left[\begin{array}{c} \dot{u}+q\cdot w\\ \dot{w}-q\cdot u\\ \dot{q} \end{array}\right]$$ (1)

$$\left[\begin{array}{c} \dot{x}\\ \dot{z}\\ \dot{\theta } \end{array}\right]=\left[\begin{array}{c} R \end{array}\right]\left[\begin{array}{c} u\\ w\\ q \end{array}\right]$$ (2)

#### 3.1.3 Flight-testing

The designs in Figures 4A,B were manufactured with the aim of performing indoor gliding tests and a total of 16 trials at 4 different release angles (25°, 35°, 45°, 55° and pitch down) were conducted for each of the designs. These trials show little dependence of the gliding trajectory on the glider’s release conditions, which is advantageous when releasing them from an UAV where there is little control over said conditions. Throughout these experimental trials, two different flight modes can be observed: a straight gliding motion and a spiral downwards motion, which was expected, since the root locus diagram generated by the stability analysis in XFLR5 indicated a spiral mode. This variability in gliding behavior is likely a direct consequence of slight variations in manufacturing or warping of the glider with changes in humidity, which can create slightly asymmetrical dihedral or changes in the center of gravity position. Furthermore, outdoor tests of nine design 4 gliders have been conducted to validate the method for real-world glider deployment. The tests have shown, that placing the glider in a natural environment–under the influence of wind–a forward gliding behavior, a mixed gliding and spiraling behavior, and pure spiraling behavior is observed. These movements follow the same behavior seen in the seeds of the Javan cucumber seed in nature. While the forward gliding motion can carry the glider further, the spiral motion acts as aerial breaking, landing the glider close to the launch position. A figure summarizing the results of the outdoor tests is given in Supplementary Figure S7. The figure shows that the spiraling was induced by the changing wind conditions in-flight. Due to the winds appearing from the right to the left side, we recorded a majority of the gliders having landed on the left side relative to the launching point. Therefore, there is strong evidence, that the wind conditions influence the flight trajectory and can trigger spiraling behavior. In fact, gust speeds of 2.4 m/sec correspond to more than 70% of the glide speed determined by the simulation, which is far above the safe operating range of a standard aircraft and affects stability in flight. The observed combined motion of gliding and spiraling during a full scale deployment mission would cover a wider area around a UAV’s path, than what a single behavior would achieve. Representative videos of the gliders’ behavior, indicating the gliding and spiraling motion in an indoor and outdoor setting, are given in Supplementary Movie S1.

**FIGURE 5:**
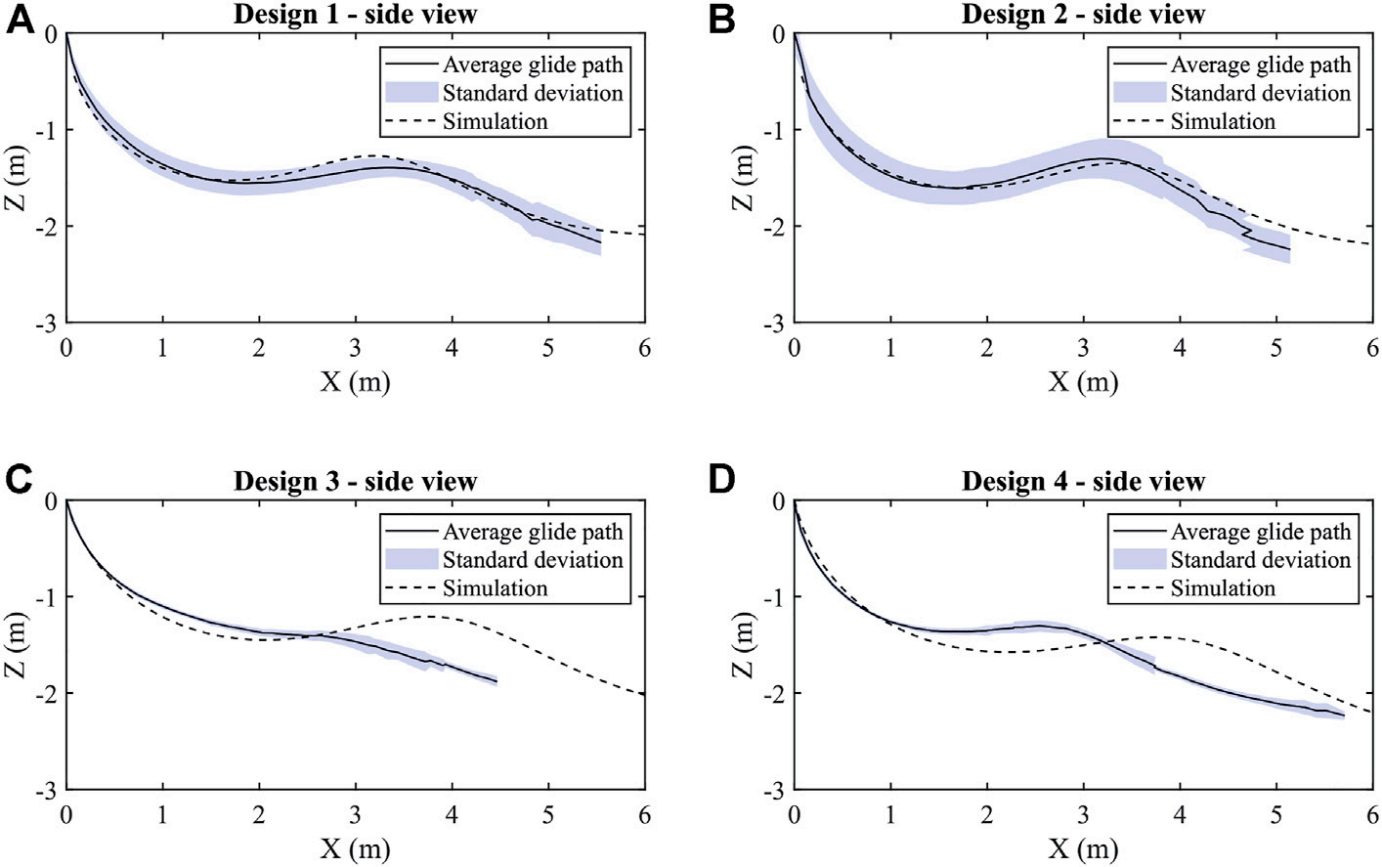
Flight paths of the two different shaped gliders with each two different centers of gravity compared with the predictions of the dynamic simulations. **(A)** gives the averaged flight-path, the standard deviation and the simulation results for the glider design 1, **(B)** shows the averaged flight-path, the standard deviation and the simulation results for the glider design 2, **(C)** summarizes the averaged flight-path, the standard deviation and the simulation results for the glider design 3, **(D)** indicates the averaged flight-path, the standard deviation and the simulation results for the glider design 4.

Even if the spiraling motion is a coupled lateral-longitudinal dynamics behavior, the forward gliding angle is the metric that limits the maximum glider spread area, and thus the analysis was focused on it. Forward gliding results for flight-tests and dynamic simulations are shown in Figure 5, from where gliding angles are extracted and compared alongside theoretical values in Table 2. In general, the simulations correctly represent the behavior of the gliders, but predict better gliding properties mainly in the design 3 and 4 cases. This is due to the presence of more sideslip and 3D effects in the trajectory of these gliders. Nevertheless, these simulations can be used as a tool to predict to maximum range of these gliders as a function of the UAV’s travel height. For example, according to the simulation glider 1 to achieves up to 50 m travel distance if launched from 10 m height, or 270 m if launched from 50 m. However, one should keep in mind that, as shown by preliminary outdoor flight tests, higher payloads are necessary to overcome wind disturbances for any length of forward gliding to occur before the glider descends into a spiral dive.

**TABLE 2:** Overview of the gliding angles determined in XFLR5, CFD, dynamic simulation and experiments.

Glider	Angle XFLR5 [°]	Angle CFD [°]	Angle simulation [°]	Angle experiment [°]
Design 1	8.31	6.28	10.62	9.49
Design 2	7.77	6.21	10.60	10.32
Design 3	7.78	6.55	11.55	13.58
Design 4	7.85	7.16	12.67	12.53

### 3.2 Humidity actuator

The micro-structure, humidity-responsiveness, water-barrier properties as well as the mechanical properties of the developed humidity-responsive material are investigated.

#### 3.2.1 Micro-structure

**FIGURE 6:**
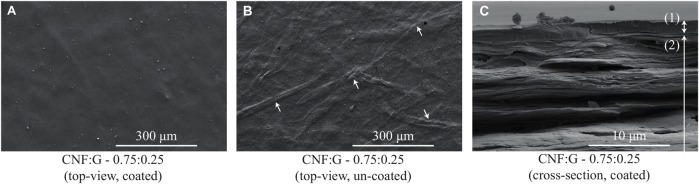
SEM images of the CNF:G—0.75:0.25 films produced at an accelerating voltage of 5 kV. **(A)** shows the top view of the CNF:G film coated with shellac, **(B)** gives the top-view of the CNF:G film without the shellac coating. The arrows indicate fibers that are visible within the composite film matrix, **(C)** shows a cross-sectional view of the CNF:G film illustrating the approximately 1.5 *μ*m coating. Label (1) indicates the thickness of the coating, while label (2) indicates the CNF:G composite system.

The micro-structure of the films and the interaction with the shellac coating was visually investigated performing scanning electron microscopy (SEM). The images were produced using a Nova NanoSEM (Nova NanoSEM 230 instrument, FEI, Hillsboro, Oregon, United States) at an accelerating voltage of 5 kV. The samples were sputtered with 7 nm platinum (BAL-TEC MED 020 Modular High Vacuum Coating Systems, BAL-TEC AG, Liechtenstein). For the cross sectional views, the samples were frozen using liquid nitrogen and then manually broken. The SEM images of the CNF:G—0.75:0.25 films are illustrated in Figure 6. Figure 6A indicates the top view after coating with shellac solution, while Figure 6B shows the same film without the coating. It can be seen that the coating reduces the surface roughness and covers smaller imperfections within the CNF:G film. Figure 6C shows the cross-sectional view of the coated film indicating the approximately 1.5 *μm* thick shellac coating and sufficient surface bonding. SEM images of the CNF:G—0.50:0.50 and CNF:G—0.25:0.75 films are given in Supplementary Figure S3. The figure summarizes the film’s top-view before and after coating as well as the cross-sectional view after coating. Additionally, the top-views of the plain films (CNF:G—0.00:1.00 and CNF:G—1.00:0.00) are given in Supplementary Figure S4.

#### 3.2.2 Humidity-responsiveness

**FIGURE 7:**
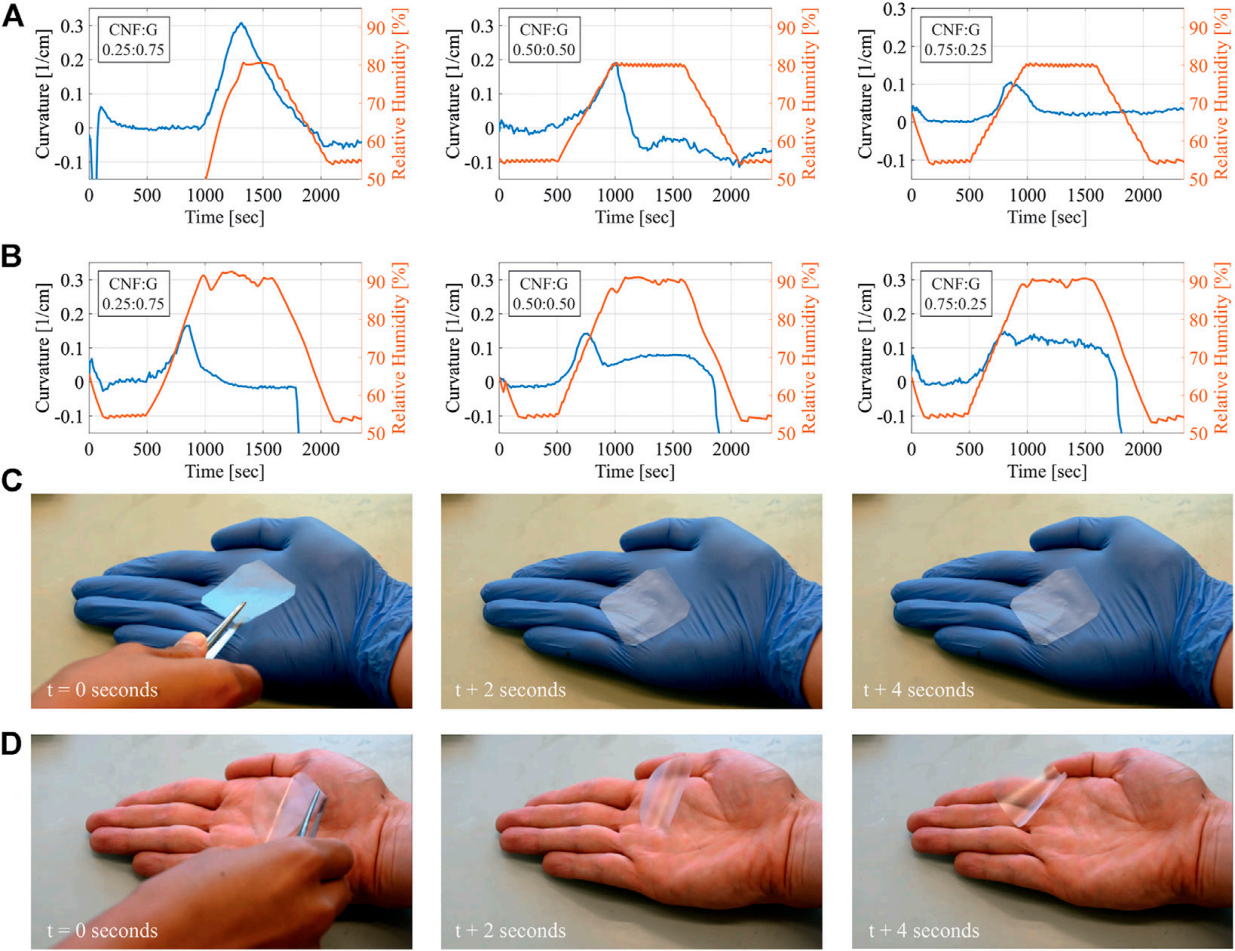
Summary of the humidity responsive film tests. To achieve quantitative values the curvature of a 1 cm by 3 cm large film was measured, while the relative humidity was varied from 55% to 80% and from 55% to 90% at a constant temperature of 20°C. Additionally, the film’s ability to react to humidity coming from the skin was qualitatively visualized. **(A)** gives the curvature versus relative humidity of the CNF:G—0.25:0.75, CNF:G—0.50:0.50 and CNF:G—0.75:0.25 film between 55% and 80%, **(B)** gives the curvature versus relative humidity of the CNF:G - 0.25:0.75, CNF:G—0.50:0.50 and CNF:G—0.75:0.25 film between 55% and 90%, **(C)** gives a photograph of the CNF:G—0.25:0.75 film placed on a hand wearing a glove after 0, 2 and 4 s, **(D)** gives a photograph of the CNF:G—0.25:0.75 film placed on a bare hand after 0, 2 and 4 s.

To evaluate the humidity-responsiveness of the actuator films, the composite films were tested using a climatic chamber (3523/16, Feutron Klimasimulation GmbH, Langenwetzendorf, Germany). Rectangular films with an aspect ratio of 1:3 were clamped in a FDM printed block and held at a fixed position in the chamber. The actuator as well as the camera, which is set to continuous shooting, were additionally covered using a mosquito screen to reduce the effect of internal air circulation on the bending of the film. The relative humidity was linearly changed from 55% (9.51 *g*/*m* ^3^) to 60% (10.374 *g*/*m* ^3^), from 55% to 70% (10.374 *g*/*m* ^3^), from 55% to 80% (13.83 *g*/*m* ^3^) and from 55% to 90% (15.56 *g*/*m* ^3^) at constant 20°C and pictures were taken every 6 s. By using a Matlab computer-vision script, the curvature of the actuator was extracted from each frame and calculated. The three different composite films (CNF:G—0.25:0.75, CNF:G—0.50:0.50, CNF:G—0.75:0.25) were analyzed. The results of the time-dependent curvature and the conditions in the climatic chambers for the runs from 55% to 80% and 55%–90% humidity are summarized in Figure 7. By comparing the films tested from 55% to 80% relative humidity, it shows that with increasing gelatin content, the curvatures of the films dramatically increase. The maximum curvatures of the films is achieved at approximately 80% relative humidity, which are 0.31 1/*cm* for CNF:G—0.25:0.75, 0.19 1/*cm* for CNF:G—0.50:0.50, and 0.11 1/*cm* for CNF:G—0.75:0.25, respectively. A video of the CNF:G—0.25:0.75 film taken inside the climatic chamber at a run from 55% relative humidity to 90% is shown in Supplementary Movie S2. The behavior is caused by water molecules intercalating between gelatin chains and disrupting inter-chain hydrogen bonds formed by amide groups at high relative humidity D’Agostino et al. (2017); Liu et al. (2021). The intercalation allows the active layer of CNF:G to absorb more water and therefore swell more than the water-resistant Zhang et al. (2020) side, leading to the film bending towards the shellac-coated side. However, the gelatine polymer matrix within the film loses its initial self-assembled arrangement and thus the actuator tends to bend back to the other side.

As a partially crystalized nanofiber, CNF has a more stable structure than gelatin polymers, which enables it to serve as a nano-support to the matrix and enhance the water containability of the actuator Sofla et al. (2016). As the relative humidity increased from 55% to 90%, Figure 7B reveals that the actuating property of CNF:G—0.75:0.25 is more stable than those with lower CNF contents. Further tests of the actuator films performed at lower relative humidity (55%–60% and 55%–70%) have shown only minor or no actuator response. Therefore, the sensitivity of the composite films is above 70% relative humidity at 20°C. The tests performed at lower humidities are summarized in Supplementary Figure S5. Qualitative tests of the uncoated films showing how they respond to skin moisture were carried out and are shown in Figure 7C and Figure 7D. To do so, a CNF:G—0.25:0.75 film was positioned on a hand wearing a rubber glove. Due to no apparent humidity gradients, the actuator did not change its shape. When placed in a hand without the glove, the actuator instantly started to coil away from the hand and move. The video of this test is given in Supplementary Movie S3.

#### 3.2.3 Water-barrier properties

**FIGURE 8:**
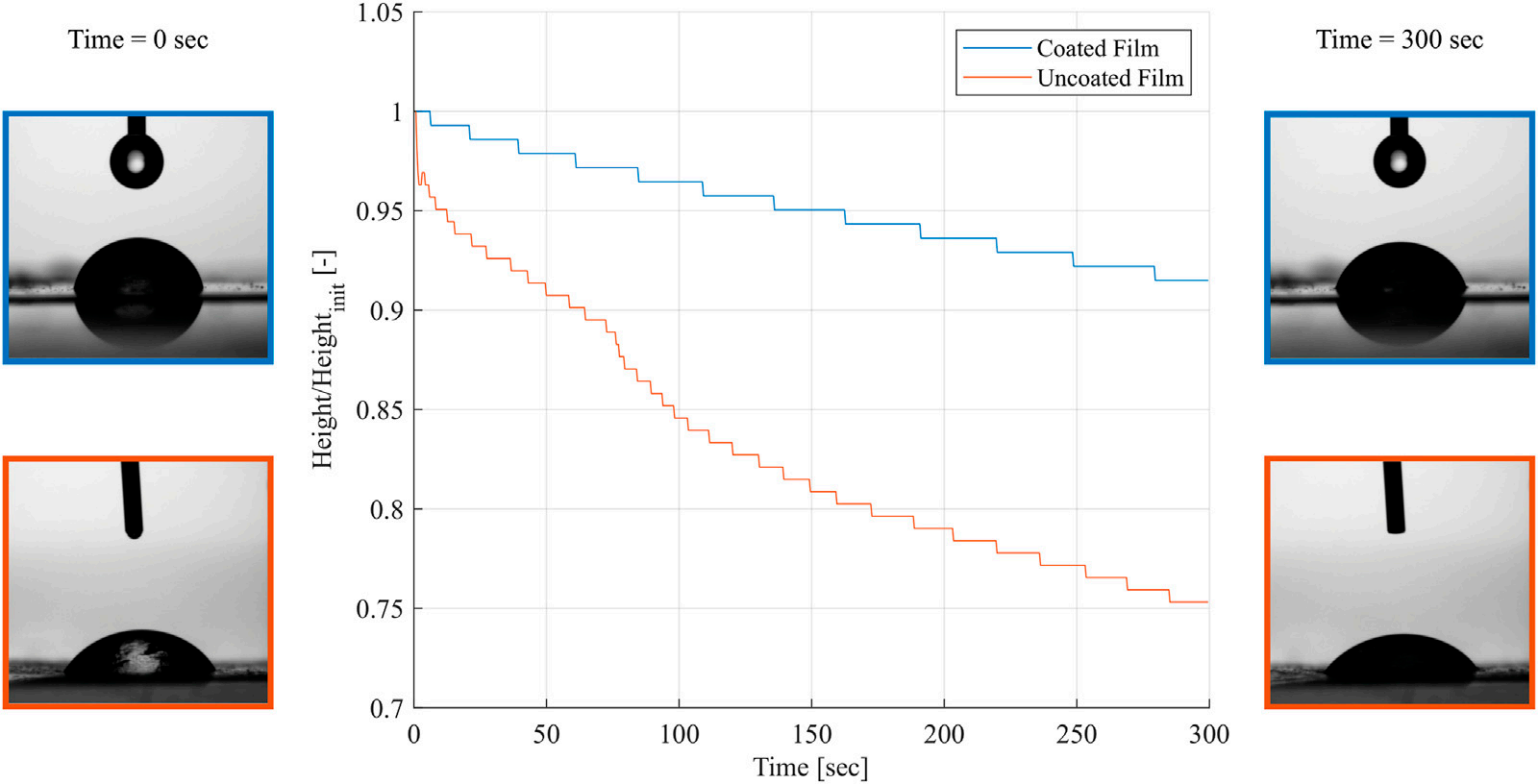
Representative time-dependent contact angle measurements of the coated and un-coated CNF:G—0.25:0.75 films for 5 min. The water droplet on the coated film shows only minor reduction in height, while the droplet on the un-coated film penetrates the film quicker.

To validate the waterproofing properties of the shellac coating, contact angle measurements were performed using a contact angle analyzer (Contact Angle System OCA, DataPhysics Instruments GmbH, Fliderstadt, Germany). The results are summarized in Figure 8. The shellac coating of the CNF:G—0.25:0.75 was found to form a waterproof layer. While the water droplet on the un-coated film reduces its height by 24.69% after 5 min, the water droplet on the coated film reduces its height by only 8.51% in the same time. It needs to be noted that some reduction of the height is due to evaporation.

#### 3.2.4 Mechanical properties

**FIGURE 9:**
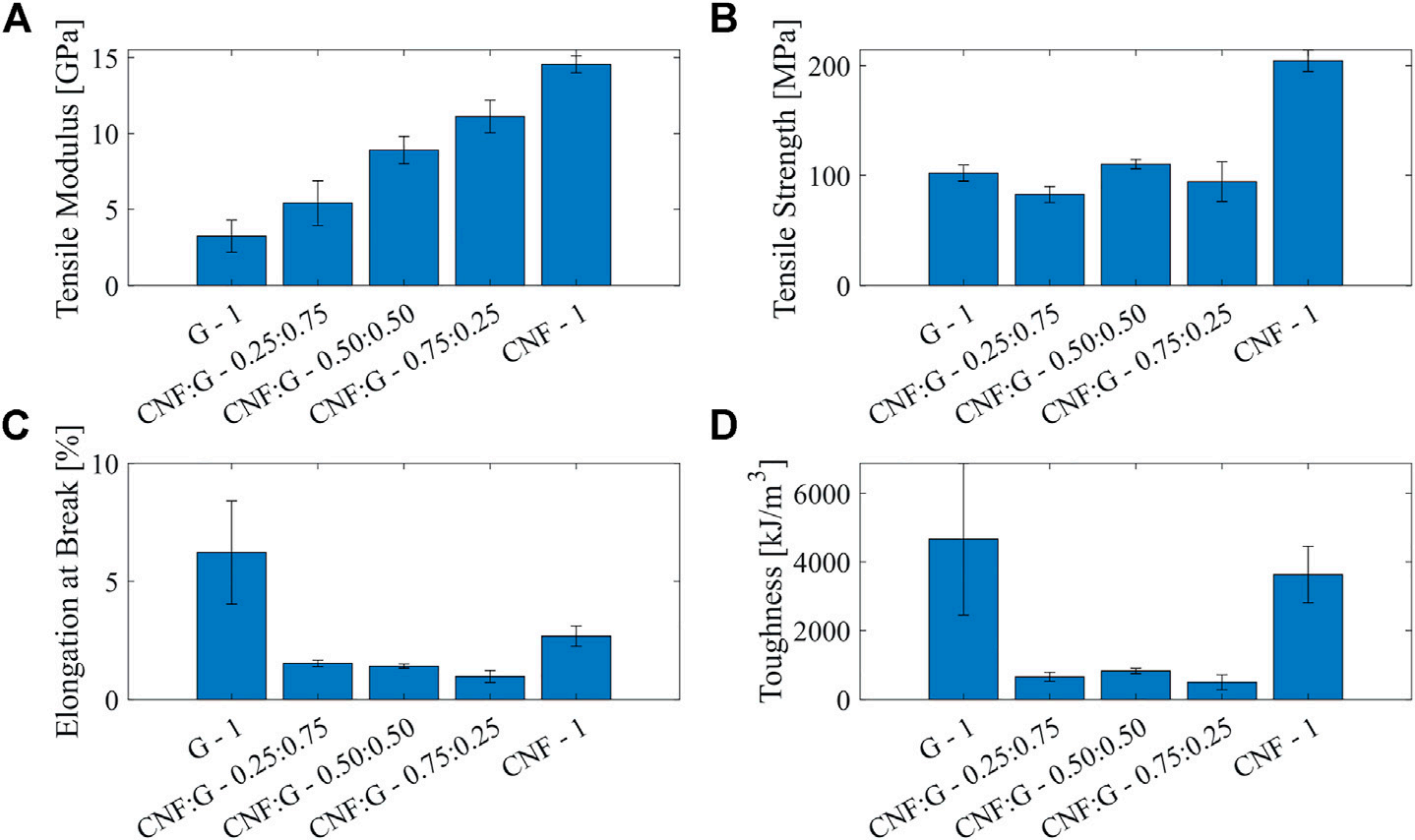
Results of the mechanical characterization of the humidity-responsive films composed G—1, CNF:G - 0.25:0.75, CNF:G—0.50:0.50, CNF:G—0.75:0.25 as well as CNF—1. **(A)** gives the tensile moduli of the films, **(B)** gives the tensile strength, **(C)** gives the elongation at break, **(D)** summarizes the tensile energy to break or toughness.

The mechanical tests were performed based on ASTM D882 ASTM (2018). The samples were tested using a universal tensile testing machine (Z010 RetroLine, ZwickRoell GmbH & Co. KG, Ulm, Germany) with a 5 kN load-cell. Five 100 mm × 10 mm large samples were cut out from the approximately 30 *μ*m thick sheets of each composite film using a CO2 laser cutter (Nova24 60 W, Thunder Laser Tech Co., Ltd., Shatian, China). The specimens were clamped using manual grips at an initial distance of 70 mm and pulled at a rate of 7 *mm*/min. The Young’s modulus was calculated by taking the slope of the stress and strain curve and the toughness was calculated by integrating the area underneath the curve. The tensile strength and the elongation at break were taken directly from the produced curve. The mechanical properties are summarized in Figure 9. It was found that the stiffness increases linearly with the CNF content within the films, ranging from 3.2 GPa up to 14.6 GPa. The tensile strengths were found to be in the range of 82.6 MPa–110.2 MPa, except for the pure CNF films that were outperforming the other films reaching values up to 204.4 MPa. The elastic plain gelatin films reached higher values for the elongation at break and toughness (6.2% and 4′661 *kJ*/*m* ^3^), while the other composite films all achieved a similar range of 0.98%–1.53% and 504.8 *kJ*/*m* ^3^ to 830.8 *kJ*/*m* ^3^, respectively. The CNF films instead reached slightly higher values (2.68% and 3′629 *kJ*/*m* ^3^). The mechanical test results are in line with their humidity-responsiveness. Gelatin acts as a polymer matrix to give the actuator a rapid response and the capability of elastic deformation, while partially crystallized CNF serves as a nano-support to enhance the stability of the structure at higher humidity.

### 3.3 Integrated glider prototype

**FIGURE 10:**
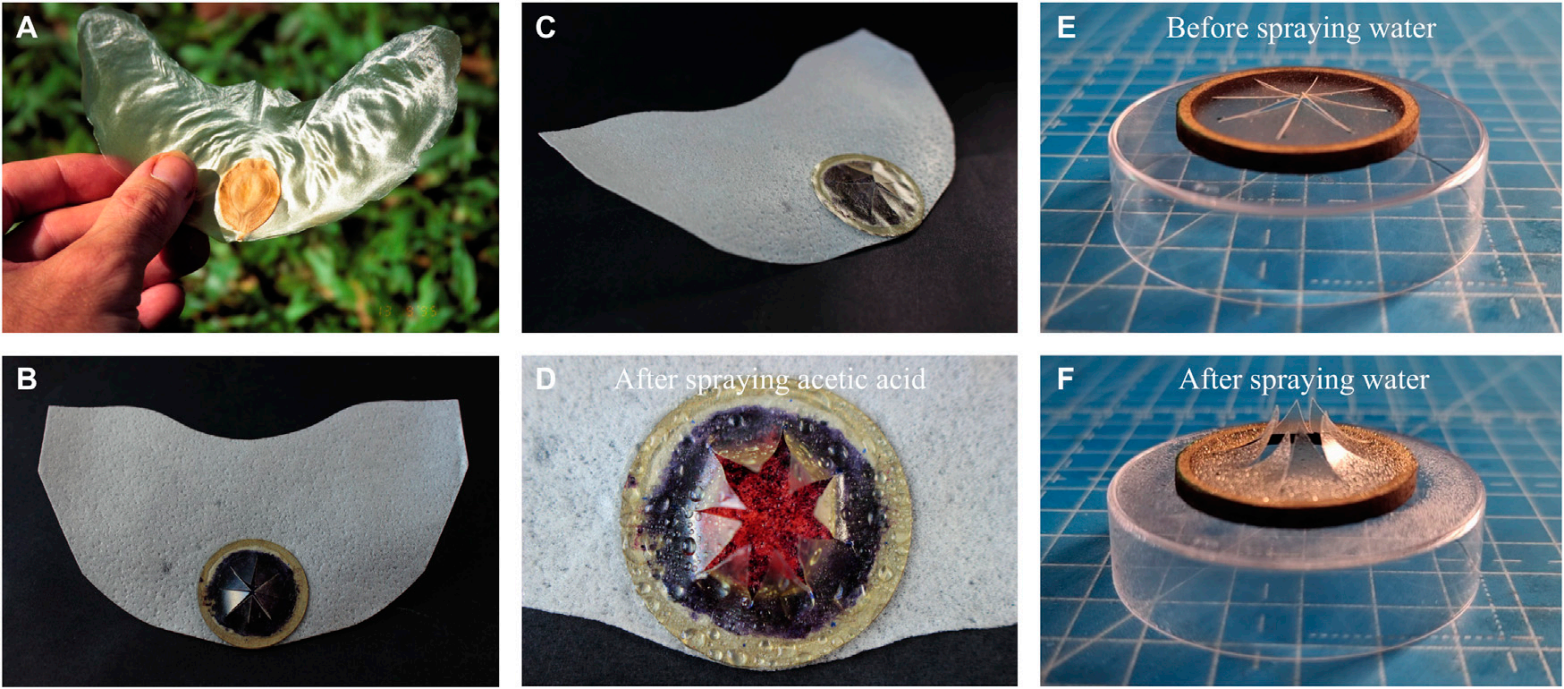
Javan cucumber seed glider template, the manufactured prototypes and the integrated actuators. **(A)** gives an image of an *Alsomitra macrocarpa* seed (CC by 2.0, photo by Scott Zona) that was used as a template for the glider, **(B)** shows the top view of one fabricated prototype with an integrated sensing layer and actuator, **(C)** shows the same prototype from a side view visualizing the 3D shape of the wing, **(D)** gives a detailed view of the actuator and sensor after it was sprayed with acetic acid, **(E)** illustrates an actuator made from CNF:G—0.25:0.75 films before it was manually sprayed with water and, **(F)** shows the same actuator after its been sprayed with water.

A picture of the biological template–the Javan cucumber seed or *Alsomitra macrocarpa*–is given in Figure 10A. The final prototype glider featuring the computed airfoil shape as well as the integrated actuator and pH sensing layer is given in Figures 10B,C. Figure 10D shows a wet actuator with an integrated sensing layer sprayed with diluted acetic acid. The color change from blue to red can be seen appearing in the exposed area of the actuator. Figures 10E,F indicate the star-shaped actuator cut from a CNF:G—0.25 : 0.75 before and after manually spraying it with water. It needs to be noted that after 30 min of ambient drying, the actuator closes fully again and therefore the movement is completely reversible. A video of the actuator sprayed with water is given in the Supplementary Movie S4.

### 3.4 Glider biodegradation

**FIGURE 11:**
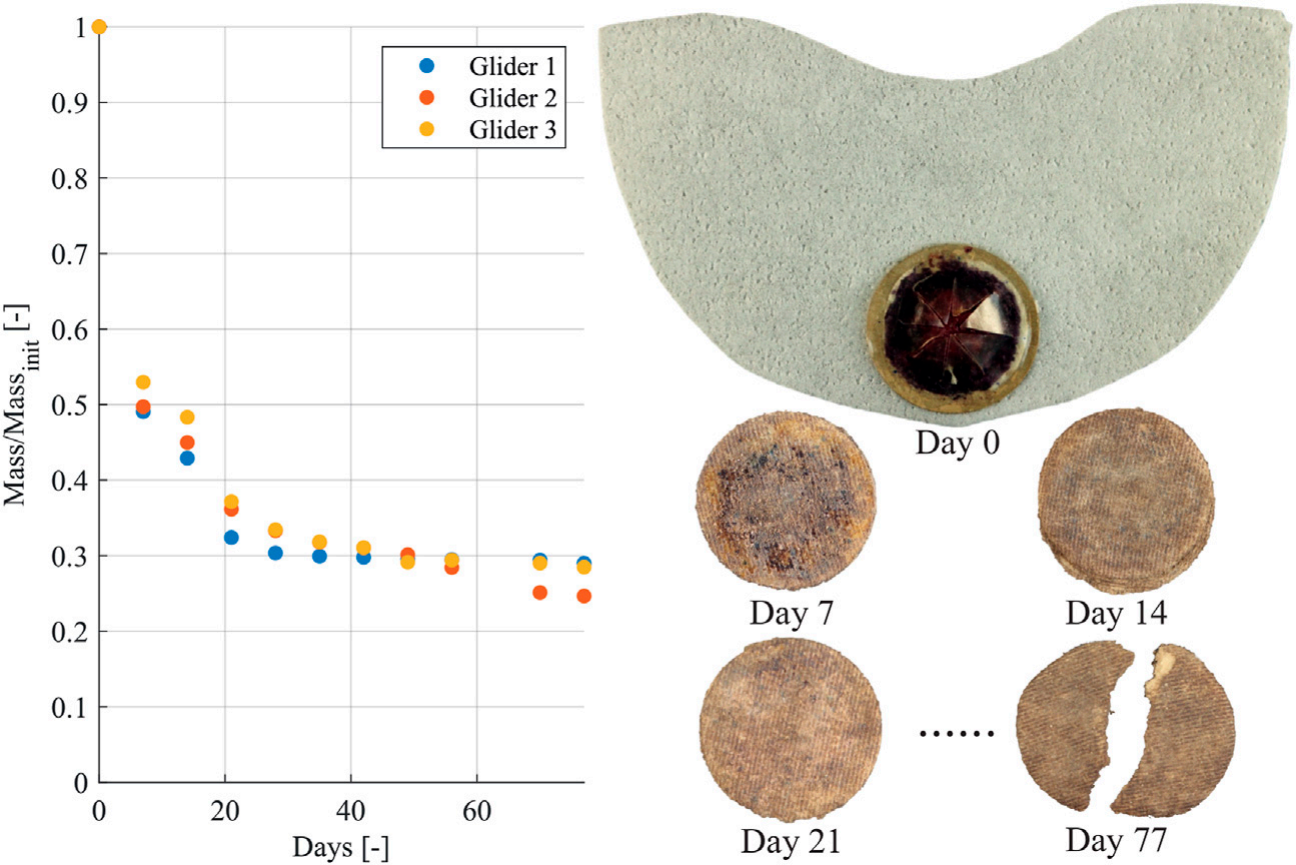
The weight loss of the glider during 77 days is shown. For the week 0, 1, 2, 3 and 11 an image of the first glider is given. Note that the glider’s wing completely degrades after 7 days of disintegration.

Biodegradation tests were performed to evaluate the rate of degradation under accelerated conditions. The norm ISO 20200 was followed in a laboratory-scale test to evaluate the disintegration rate of the glider under simulated aerobic composting conditions. The tests were performed for 77 days at constant 58°C. The soil simulator was composed of sawdust, rabbit feed, cornstarch, sugar, corn oil, urea and compost. The sample was put inside a protective mesh made from polypropylen and buried completely in the soil. The mass of the sample was then weighed with and without the protective mesh in week 1, 2, 3, 4, 5, 6, 7, 8, 10 and 11. Figure 11 indicates the weight loss over time and gives images of the glider after the first, second, third and 11th week of degradation. After the first week the glider air-frame has already completely degraded, while the actuator as well as the actuators cellulose substrate need more time for disintegration. SEM images of the potato starch based wafer papers are given in Supplementary Figure S6. The high porosity is one of the main factors enabling its fast degradation.

## 4 Discussion

This paper presents the design, fabrication, and performance evaluation of *Alsomitra macrocarpa* seed inspired transient gliders with embodied humidity responsive actuators. The transient aerial robot, designed for rainwater pH analysis, highlights gliding abilities with a gliding ratio of up to ∼ 10° and degrades at a fast rate–the wing disintegrates fully within 1 week. The humidity actuator embedded in the glider is also used as a payload that adjusts the CG for optimal flight performance. It was shown that, the flight characteristics of the chosen airfoil, exceed the ones of the averaged Javan cucumber seed as well as the flat plate with and without a flap. The proposed simulation frame work, has guided towards the design of a suitable glider configuration, outperforming the biological template. Furthermore, the actuators feature an opening and closing mechanism that is highly sensitive to relative humidity changes from 70% and reactivity to direct water mist exposure. When these activation conditions are met, the actuators opens, allowing selective access to the pH sensor underneath.

This work has demonstrated the ability of the gliders to disperse around a UAVs flight-path, for future use in environmental monitoring. While the forward gliding mode helps the glider to carry the sensor forward further distances, the spiral mode narrows down its landing position; which can result in a more homogeneous distribution of the sensors even when deployed from the same starting point. Initial deployment trials featuring a quad-rotor platform have been conducted successfully. An image of the developed platform is given in Supplementary Figure S8 and a video of a glider deployment is found in the Supplementary Movie S5. However, further theoretical and experimental work is necessary to predict the properties of this distribution. For example, a coupled longitudinal-lateral dynamics simulation can create insight into what level of disturbance is necessary to trigger a spiral dive in different glider designs. Additionally, computer vision methods could be deployed to track the glider after deployment from the drone to further estimate the landing site and therefore achieving quicker data retrieval from the sensor after rain-water analysis. To accomplish sufficient pH resolution, a calibration curve of the litmus based sensors needs to be acquired. The RGB values, which are visually collected from the sensors, are then matched with the curve and converted into a pH value.

In this work, manufacturing inconsistencies between manufactured gliders in the molding process were noticed. These are due to the humidity in the air, the amount of sprayed water prior to mold pressing, drying time, and the clamping force of the molds. These all played a role in the final geometry of the glider, affecting the glider’s dihedral, airfoil camber, and asymmetric features. To monitor these inconsistencies and evaluate variability in manufacturing, one can utilize laser scanners to get accurate 3D models of the gliders. This would allow to create a probability distribution of glider geometrical parameters and consequentially predict flight behaviors. Furthermore, encapsulating the wing with a water-resistant coating could help to mitigate the deformations introduced by storing the glider in ambient humidity conditions.

In the future various approaches could be facilitated to manufacture biodegradable sensors capable of measuring other environmental parameters–such as relative humidity, temperature, UV intensity and micro pollutant levels. Those sensors can then be integrated on the proposed glider design enabling remote sensing of multiple ecological variables.

## Data Availability

The original contributions presented in the study are included in the article/Supplementary Material, further inquiries can be directed to the corresponding authors.
