# Erratum: Transient bio-inspired gliders with embodied humidity responsive actuators for environmental sensing

**DOI:** 10.3389/frobt.2023.1173498

**Published:** 2023-03-07

**Authors:** Frontiers Production Office

**Affiliations:** Frontiers Media SA, Lausanne, Switzerland

**Keywords:** biodegradable materials, transient robotics, environmental sensing, aerial robotics, bio-inspiration

An omission to the **Funding** section of the original article was made in error. The following sentence has been added: “Open access funding was provided by Empa - Swiss Federal Laboratories For Materials Science And Technology.”

The original version of this article has been updated.

