# Supplementary material for: Transient bio-inspired gliders with embodied humidity responsive actuators for environmental sensing
Source: Front Robot AI. 2022 Oct 31;9:1011793. doi: 10.3389/frobt.2022.1011793 (PMC9659746; doi:10.3389/frobt.2022.1011793)
Supplement: Supplementary file 2 [file DataSheet1.pdf]

Supplementary Material

1 SUPPLEMENTARY DATA

1.1 Movies

1.1.1 Supplementary Movie S1

Supplementary material S1 highlights representative flight tests of the type 1 and type 2 glider performed in the flight arena. Furthermore, demonstration of the glider performance outdoor is shown. The video indicates a mixed spiraling and gliding behavior, which is more commonly found outdoors due to the effect of the wind.

1.1.2 Supplementary Movie S2

Supplementary material S2 shows a frame-by-frame movie of a CNF:G 0.75:25 film, which is placed inside the climatic chamber. The relative humidity was changed from 55% to 90 %, held for 10 minutes and reduced to 55 % relative humidity at constant 20 °C. This video was used as an input video to extract the curvature during the actuator characterization.

1.1.3 Supplementary Movie S3

Supplementary material S3 shows a film made from CNF:G 0.75:25 placed on a bare hand. Afterwards, the film is placed on a hand that is covered by a glove. It can be seen that the film reacts by curling up to the humidity that comes from the hand's humidity.

1.1.4 Supplementary Movie S4

Supplementary material S4 shows a star-shaped actuator made from CNF:G 0.75:25. It is manually sprayed with water using a nebulizer. It can be seen that the actuator opens after it get sprayed with the water mist.

1.1.5 Supplementary Movie S5

Supplementary material S5 shows a video of an indoor deployment of two gliders using a quad-rotor platform.

1.2 Supplementary Figures and Tables

1.2.1 Supplementary Figure S1

Supplementary figure S1 shows the plots of the convergence study performed.

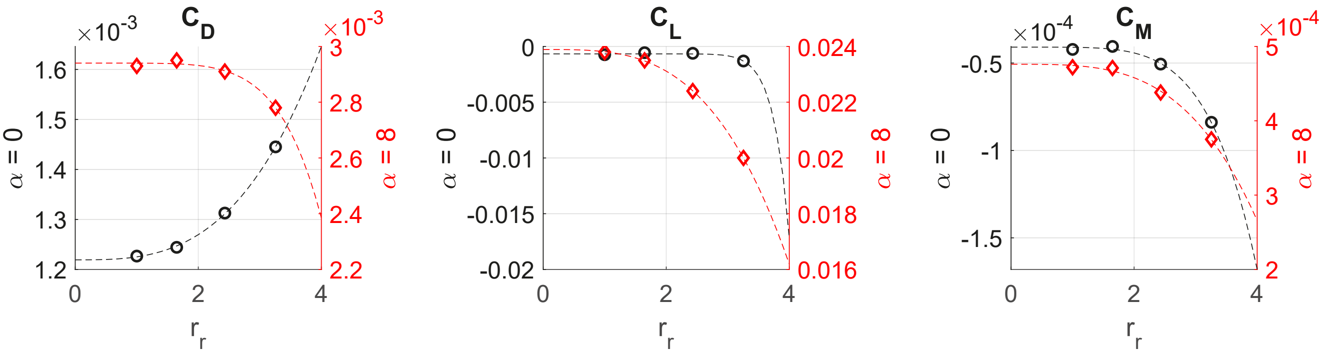

Figure S1. Convergence of the lift, drag and moment coefficients for increasing mesh refinement levels depending on the refinement ratios. Discretisation error is estimated by least-squares fitting of a truncated power series.

1.2.2 Supplementary Figure S2

Supplementary figure S2 shows the flow-field around the glider in longitudinal section (mid-span) at $8°$ AoA as well as the flow-field in transverse section (mid-cord) at $10°$ AoA. The color corresponds to the velocity magnitude in m/s.

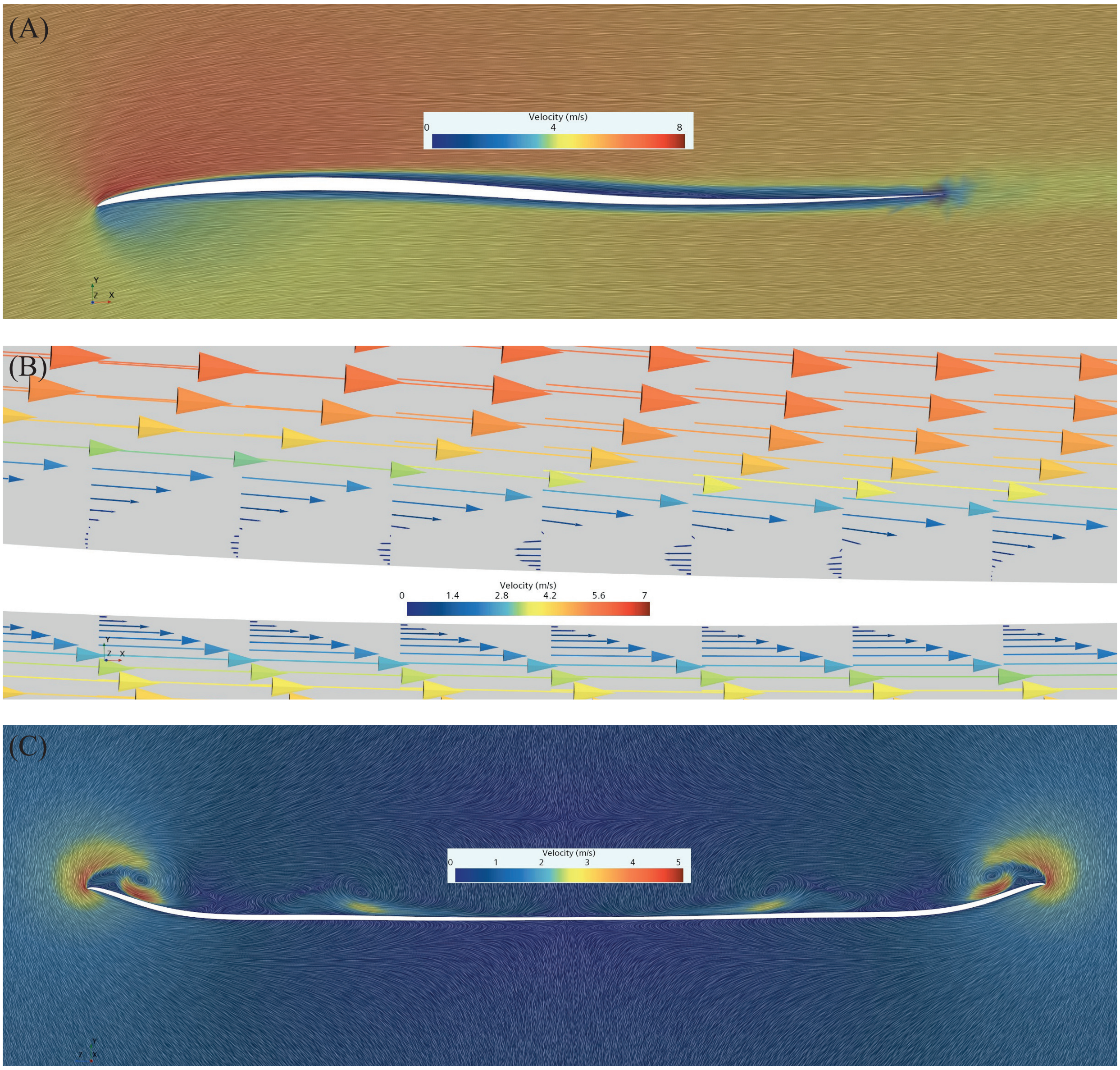

Figure S2. CFD visualizations of the flow at 8 ° AoA and at 10 ° AoA. **(A)** gives the flow-field around the glider in longitudinal section (mid-span) at 8 ° AoA, **(B)** shows a detailed view of the same flow-field of the separation bubble at mid-cord position, **(B)** indicates the flow-field in transverse section (mid-cord) at 10 ° AoA.

1.2.3 Supplementary Figure S3

Nine gliders have been launched five times each from a 30 cm long launching slope mounted on a 6.9 m high bridge at an angle of $45°$. The landing site of the gliders was measured using a tape measure and a plot was generated from the results. During the testing crosswinds at the launching position were measured using a hot-wire anemometer (testo 405i, Testo SE & Co. KGaA, Titisee-Neustadt, Germany). The average cross-wind speed during testing was $0.53\ m/sec$ and the 20 second gust-speed was found to be $2.4\ m/sec$. Supplementary figure S7 summarizes the results.

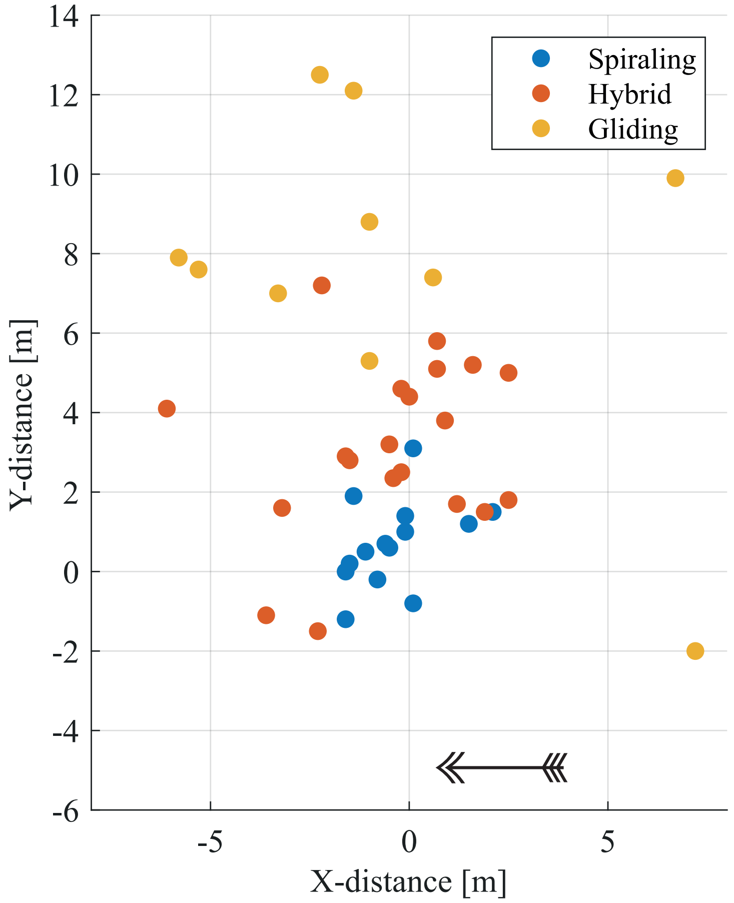

Figure S3. Landing positions relative to the launching point of 45 launches. The experienced wind-speed direction is indicated by an arrow on the lower right corner. The experienced wind-speed direction is indicated by an arrow on the lower right corner. Gliding, spiraling and a hybrid behavior were observed during testing.

1.2.4 Supplementary Figure S4

Supplementary figure S4 shows SEM images of the CNF:G - 0.50:0.50 and CNF:G - 0.25:0.75 films before and after coating.

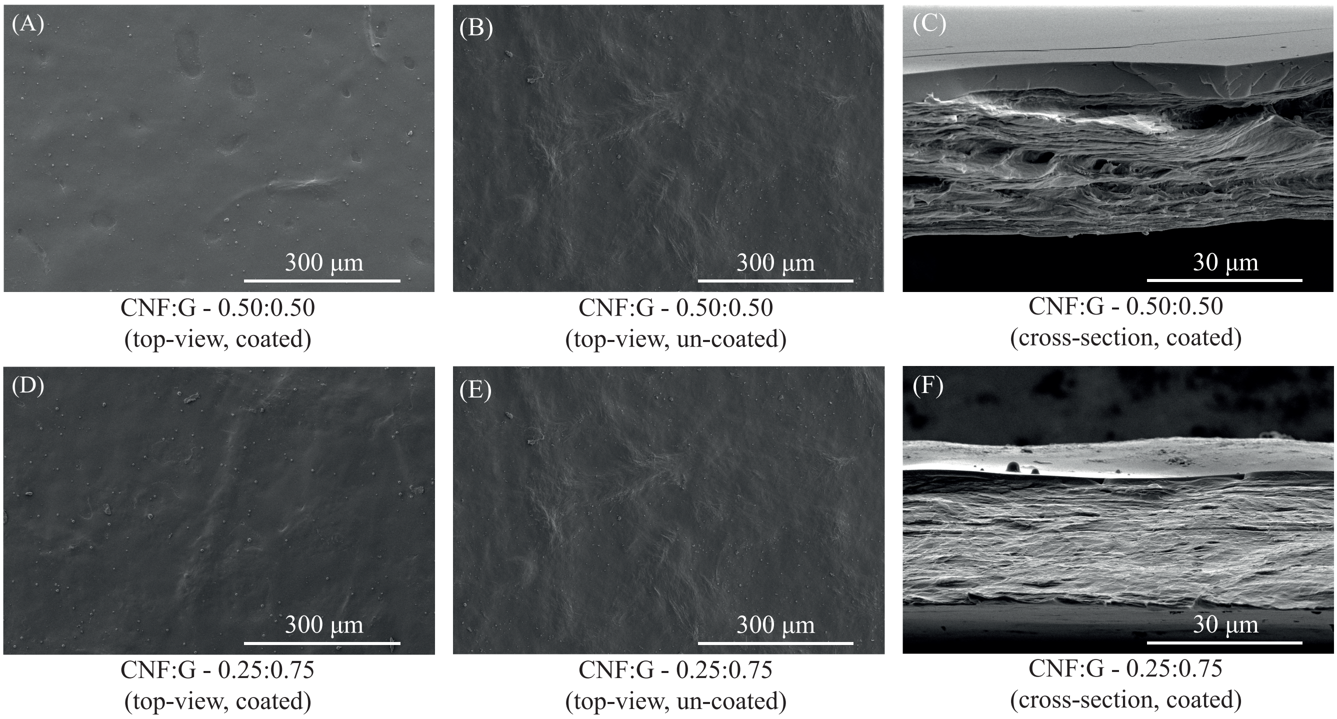

Figure S4. SEM images of the CNF:G composite films produced at an accelerating voltage of 5 kV. **(A)** shows the top view of the CNF:G - 0.50:0.50 film coated with shellac, **(B)** gives the top-view of the CNF:G - 0.50:0.50 film without the shellac coating, **(C)** shows a cross-sectional view of CNF:G - 0.50:0.50 film, **(D)** shows the top view of the CNF:G - 0.25:0.75 film coated with shellac, **(E)** gives the top-view of the CNF:G - 0.25:0.75 film without the shellac coating, **(F)** shows a cross-sectional view of CNF:G - 0.25:0.75 film.

1.2.5 Supplementary Figure S5

Supplementary figure S4 shows SEM images of the plain films manufactured from gelatin and CNF (CNF:G - 0.00:1.00 and CNF:G - 0.00:1.00).

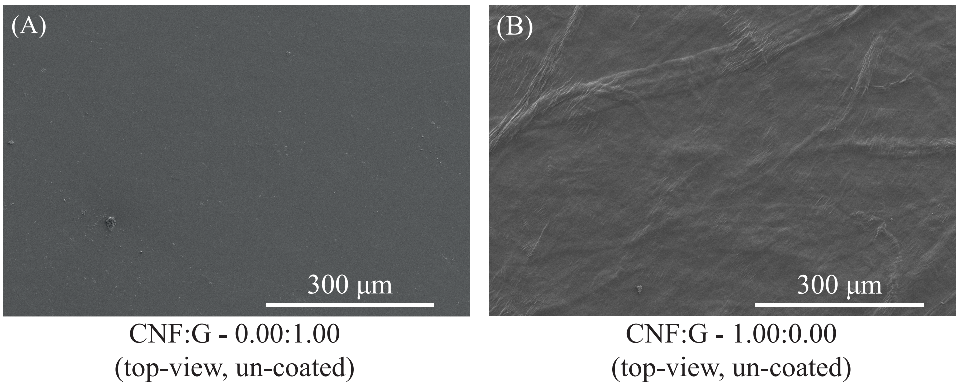

Figure S5. SEM images of the plain films produced at an accelerating voltage of 5 kV. **(A)** shows the top view of the CNF:G - 0.00:1.00 film, **(B)** shows the top view of the CNF:G - 1.00:0.00 film.

1.2.6 Supplementary Figure S6

Supplementary figure S5 shows the curvature of the humidity responsive films tested from 55 % to 60 % and from 55 % to 70 % relative humidity. While all three film compositions showed no responsiveness at 60 % relative humidity, there were only slight changes in the curvature detected while testing from 55 % to 70 %.

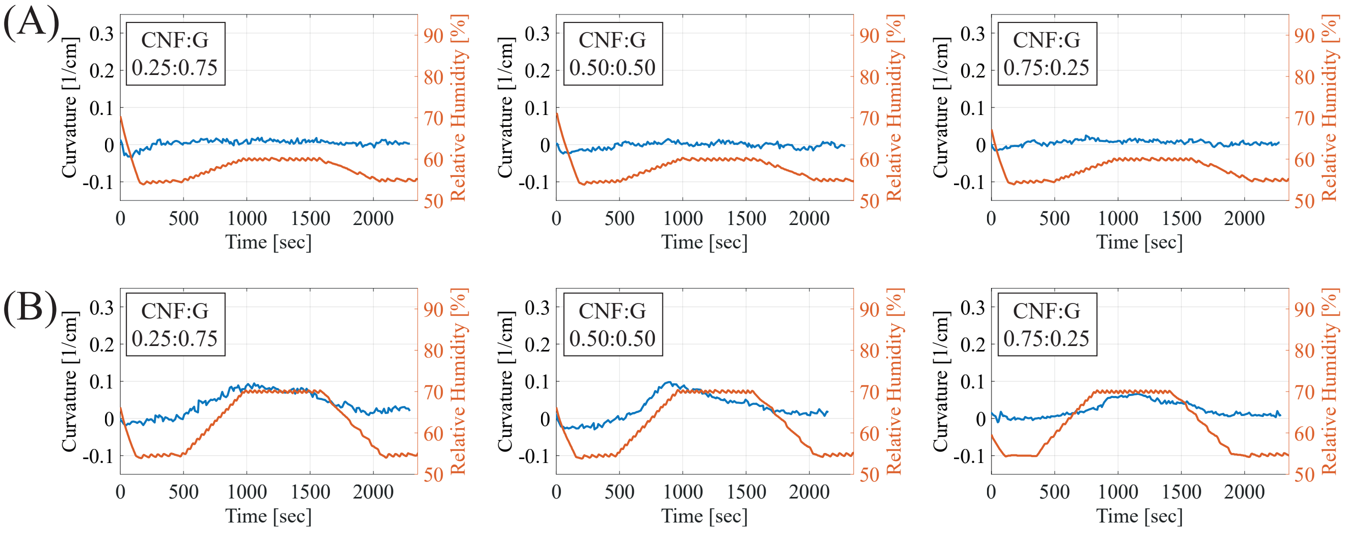

Figure S6. Summary of the humidity responsive film tests at 55 % to 60 % and 55 % to 70 % relative humidity. 1 cm by 3 cm large films were tested. **(A)** gives the curvature versus relative humidity of the CNF:G - 0.25:0.75, CNF:G - 0.50:0.50 and CNF:G - 0.75:0.25 film between 55 % and 60 %, **(B)** gives the curvature versus relative humidity of the CNF:G - 0.25:0.75, CNF:G - 0.50:0.50 and CNF:G - 0.75:0.25 film between 55 % and 70 %.

1.2.7 Supplementary Figure S7

Supplementary figure S7 shows SEM images of the porous potato starch based wafer paper that was used to form the lightweight wing structure of the glider. The porous nature of the material helped to accelerate the degradation of the glider.

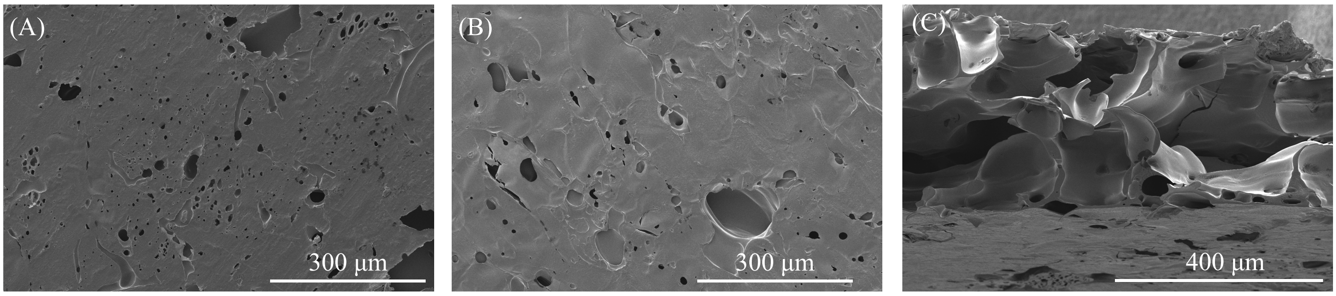

Potato starch wafer paper (front-view) | Potato starch wafer paper (back-view) | Potato starch wafer paper (cross-section view)

Figure S7. SEM images of the commercial potato starch paper used to manufacture the glider's wing produced at an accelerating voltage of 5 kV. **(A)** starch film form the front-view, **(B)** starch film from the back-view, **(C)** cross-sectional view of the film broken after freezing it in liquid Nitrogen.

1.2.8 Supplementary Figure S8

Supplementary figure S8 shows the developed quad-rotor platform capable of deploying various gliders selectively.

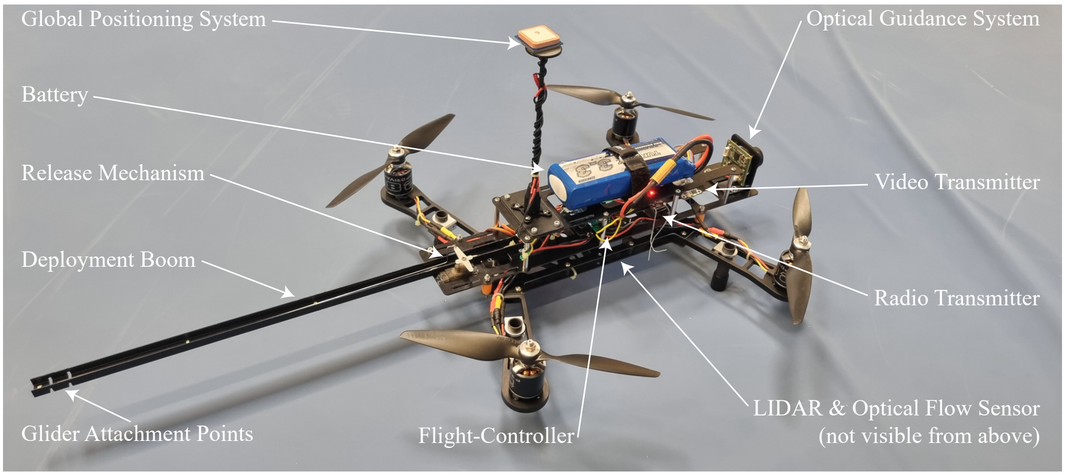

Figure S8. Quad-rotor platform with integrated glider deployment mechanisms. The robot features a live video transmission system, an optical flow and light detection and ranging (LIDAR) sensor for flight stabilization and a GPS module enabling way-point mission for future glider deployments.

1.2.9 Supplementary Table S1

Supplementary table S1 summarizes the error of the increasingly coarse meshes.

Table S1. Error of increasingly coarse meshes relative to the exact solution estimated by asymptotic convergence.

Mesh size (mill)	C_D 0°	C_L 0°	C_M 0°	C_D 8°	C_L 8°	C_M 8°
26.5	0.49 %	0.15 %	0.21 %	0.12 %	0.32 %	0.33 %
5.9	2.3 %	0.80 %	2.9 %	1.1 %	1.7 %	1.9 %
1.8	7.5 %	1.9 %	22 %	2.8 %	6.2 %	7.6 %
